# Unpacking the Black Box: A Formative Research Approach to the Development of Theory-Driven, Evidence-Based, and Culturally Safe Text Messages in Mobile Health Interventions

**DOI:** 10.2196/mhealth.4994

**Published:** 2016-01-22

**Authors:** Marion A Maar, Karen Yeates, Zsolt Toth, Marcia Barron, Lisa Boesch, Diane Hua-Stewart, Peter Liu, Nancy Perkins, Jessica Sleeth, Mary Jo Wabano, Pamela Williamson, Sheldon W Tobe

**Affiliations:** ^1^ Faculty of Medicine Northern Ontario School of Medicine Laurentian University Sudbury, ON Canada; ^2^ Department of Medicine Queens University Kingston, ON Canada; ^3^ Department of Medicine Northern Ontario School of Medicine Sudbury, ON Canada; ^4^ Riverstone Research Consulting Beamsville, ON Canada; ^5^ Department of Research Northern Ontario School of Medicine Sudbury, ON Canada; ^6^ Department of Medicine Sunnybrook Health Sciences Centre, Sunnybrook Research Institute University of Toronto Toronto, ON Canada; ^7^ University of Ottawa Heart Institute Ottawa, ON Canada; ^8^ Wikwemikong Health Centre Wikwemikong, ON Canada; ^9^ Noojmowin Teg Health Centre Little Current, ON Canada

**Keywords:** Aboriginal people, behavioral change wheel, cultural safety, grounded theory, mobile phone, semiotics, SMS, Tanzania, text messages

## Abstract

**Background:**

Mobile-cellular subscriptions have increased steadily over the past decade. The accessibility of SMS messages over existing mobile networks is high and has almost universal availability even on older and unsophisticated mobile phones and in geographic settings where wireless coverage is weak. There is intensive exploration of this inexpensive mobile telecommunication technology to improve health services and promote behavior change among vulnerable populations. However, a neglected area of research is the documentation and critical analysis of the formative research process required in the development and refinement of effective SMS messages.

**Objective:**

The objective of this qualitative research study was to identify major factors that may impact on the effectiveness of evidence-based SMS messages designed to reduce health inequities in hypertension management in low resource settings, including Aboriginal populations in high-income countries and rural populations in low-income countries. Specifically, we were interested in uncovering the range of mediators that impact on appropriate message content transmission and, ultimately, on health behavior improvements in a range of these sociocultural settings.

**Methods:**

Collaborative qualitative research with Canadian Aboriginal and Tanzanian participants was conducted to deconstruct the content and transmission of evidence-based health information contained in SMS messages in the context of an international research project designed to address health inequalities in hypertension, and to develop a grounded theory of the major factors that mediate the effectiveness of this communication. We also examined the interrelationship of these mediators with the three essential conditions of the behavior system of the Behavioral Change Wheel model (capability, opportunity, and motivation) and cultural safety.

**Results:**

Four focus groups with a total of 45 participants were conducted. Our grounded theory research revealed how discrepancies develop between the evidence-based text message created by researchers and the message received by the recipient in mobile health interventions. These discrepancies were primarily generated by six mediators of meaning in SMS messages: (1) negative or non-affirming framing of advocacies, (2) fear- or stress-inducing content, (3) oppressive or authoritarian content, (4) incongruity with cultural and traditional practices, (5) disconnect with the reality of the social determinants of health and the diversity of cultures within a population, and (6) lack of clarity and/or practicality of content. These 6 mediators of meaning provide the basis for sound strategies for message development because they impact directly on the target populations’ capability, opportunity, and motivation for behavior change.

**Conclusions:**

The quality of text messages impacts significantly on the effectiveness of a mobile health intervention. Our research underscores the urgent need for interventions to incorporate and evaluate the quality of SMS messages and to examine the mediators of meaning within each targeted cultural and demographic group. Reporting on this aspect of mobile health intervention research will allow researchers to move away from the current black box of SMS text message development, thus improving the transparency of the process as well as the quality of the outcomes.

## Introduction

Mobile-cellular subscriptions have increased steadily over the past decade with current subscription rates ranging from 69.3 per 100 inhabitants in the African region to 140.6 per 100 inhabitants in Communist Independent States [1]. The accessibility of SMS (short message service) over existing mobile networks is high. It has almost universal availability even on older and unsophisticated mobile phones and in geographic settings where wireless coverage is weak. The high penetration of SMS texting in low- and high-resource settings has resulted in intensive exploration of this inexpensive mobile telecommunication technology as a promising application in interventions to improve health services and promote behavior change among vulnerable populations [2]. Other attractive features of text messaging for health interventions include the potential for scalability of an intervention, option to tailor messages based on age, gender, and ethnicity of a target group, and ability to send time sensitive information [3].

SMS-based health intervention messages delivered to personal mobile phones have been applied to disease prevention interventions, disease treatment interventions, disease surveillance, and to track adherence [4]. Systematic reviews have found that SMS messages can positively impact on personal health behaviors including diabetes self-management, weight loss, physical activity, smoking cessation, medication adherence for antiretroviral therapy, as well as on chronic disease management and prevention in low- and middle-income countries (LMIC) by addressing access, coverage, and equity gaps in low resource settings. Much of the current research has focused on the effectiveness of mobile health interventions as a whole with respect to behavior change and clinical outcomes, but more research is required on the unique characteristics and components of interventions [5-8].

The SMS message development process in particular is a component that is commonly hidden within a black box, lacking deconstruction of the steps involved in creating messages as well as rigorous evaluations of the quality of the health communication messages. Based on their review of the literature on SMS interventions, Fitts Willoughby and Furberg conclude that text message development and pretesting should follow best practices similar to those used for health communication messages delivered through other channels like mass media [9]. They further stress that pretesting should be conducted with the target audiences and that the associated findings should be reported.

Lim and colleagues have also emphasized the need for quality, rigor, careful development and evaluation of these brief messages [10]. After careful crafting of the messages, they argue that pretesting of the messages is necessary to ensure that they are relevant and have the expected impact on the target population. Gurman and coworkers agree that the pretesting of messages should be a priority in order to understand the needs of the target audience [2]. Accordingly, published formative research has already demonstrated audience preferences for specific types of messages such as positive, encouraging, practical and concise messages [11,12].

Clearly, a neglected area in the published literature is the documentation and critical analysis of the formative research process required in the development and refinement of messages [13]. To improve future research and program implementation, this research should be accessible for scientific scrutiny, including a description of the development of the SMS content, the theoretical basis of the messages, cultural adaptation to the target population and context along with an analysis of how the content is received by users [14]. Ideally, reporting should include a rationale for the chosen theory of behavioral change, the quality of the evidence on which the messages are based, as well as the fit of the messages within the culture and context of the targeted groups [15].

We address this gap in the literature by reporting on our research process on the development and refinement of SMS messages for an international research project designed to address health inequalities in hypertension entitled DREAM-GLOBAL (Diagnosing hypeRtension - Engaging Action and Management in Getting LOwer Bp in Aboriginal and LMIC). In the DREAM-GLOBAL study, SMS messages are designed to impart evidence-based health knowledge related to hypertension management in patients in culturally and geographically diverse, low resource environments, including diverse, rural and remote Aboriginal communities in Canada and rural communities in northern Tanzania.

The objective was to conduct qualitative research to identify major factors that may impact on the effectiveness of evidence-based SMS messages designed to reduce health inequities in hypertension management in low resource settings, including Aboriginal populations in high-income countries and subsistence farmers in low-income countries. Specifically, we were interested in uncovering sociocultural factors that mediate effective communication of content, influence behavioral responses, and ultimately impact on health behavior changes in these settings.

This paper describes the formative research process applied in DREAM-GLOBAL to develop messages, their theoretical grounding, and the sociocultural mediators that had to be considered to ensure optimal effectiveness of the transmission of the content of messages and optimize their potential to encourage healthy behaviors.

## Methods

The DREAM-GLOBAL project approach is defined by community-based participatory research (CBPR) principles that include a commitment to community collaboration in all phases of the research, acknowledging the value of practical knowledge, building on existing community strengths, and integrating research and knowledge exchange to address inequalities [16-17].

All communities participating in the DREAM-GLOBAL study were invited to participate in the formative research on the development of culturally safe SMS messages. As part of the CBPR approach, recruitment of participants in each community was led by community health staff who invited a representative group of community members with hypertension or at risk of hypertension and who had recently accessed services at the local clinics. Incentives included a luncheon and modest financial compensation to cover expenses such as travel and childcare.

The Aboriginal communities within Canada were culturally, linguistically and geographically diverse and included rural and remote locations. Testing the messages in each of the three Aboriginal communities was therefore deemed to be essential. In Tanzania only one community was able to participate at the time of the formative research, however the two communities participating in DREAM-GLOBAL were culturally, linguistically and geographically quite similar. This suggested to us that one focus group would be sufficient to represent the major views and attitudes that exist in the two communities. Member checking of the emerging themes with local Tanzanian research assistants confirmed that the emerging themes represented community attitudes and realities among the subsistence farmers and small business owners in both communities very well.

### Drafting a Set of Evidence-Based Messages

As a starting point, the content of the messages was informed by the Canadian Hypertension Education Program [CHEP] clinical practice guidelines, which include healthy eating messages from the Dietary Approaches to Stop Hypertension [DASH] diet [18]. The comprehensive set of messages was created by clinician researchers (ST, KY, ZT) with excellent knowledge of the hypertension clinical practice guidelines and extensive dietetic counseling experience within the target population in Canada (ZT). The message content focused on both drug therapy and health behavior changes.

### Shaping Evidence-Based Content Into Meaningful, Culturally Safe Text Messages

A process was designed (1) to develop a grounded theory of cultural and contextual factors that may mediate the effective transmission of the evidence-based content coded into SMS messages for target audiences in Canadian Aboriginal and Tanzanian partner communities [19]; and (2) to adjust the messages through dialogue until the content of the SMS text coded by researchers and the meaning received by the target audience was highly congruent based on the focus group discussion.

To achieve this, focus groups were conducted by experienced qualitative health researchers (MB, MM, ZT), audio recorded and transcribed verbatim. In each session, participants who had some knowledge and awareness of hypertension, were shown a total of approximately 50 text messages. In Canada, focus group discussions were conducted in English. In Tanzania, however, all messages were first translated into Swahili and then presented to community members. Focus group discussions were also translated from Swahili to English, and English to Swahili in real time with the help of two skilled translators. The transcripts were back-translated by an independent translator to ensure accuracy of the translated content prior to analysis.

We applied grounded theory to guide our analysis. Grounded theory is a qualitative research approach designed to produce new substantive theory grounded within the collected data instead of analyzing the data through a predetermined theoretical framework [20]. Grounded theory is therefore particularly useful when little is known about the studied phenomenon, as was the case in our study of the major factors that mediate the effectiveness of SMS health communication with Aboriginal people in Canada and Tanzanian villagers. NVivo 9 Qualitative Research Software was used to code the data. Consistent with grounded theory, coding categories were grounded in the focus group data instead of trying to force the data into preconceived theories or categories [20]. During early analysis, the emerging themes were formally discussed with Canadian and Tanzanian project staff and collaborators as a form of member checking to ensure research rigor and culturally relevant interpretation. Final focused code categories were developed through consensus between two of the researchers (LB, MM).

### Theoretical Frameworks That Guided the Formative Research

The text messages in this project were developed for eventual application in diverse cultural environments. An important question was whether or not the content of the evidence-based messages would be received by the target population as intended by the clinical researchers. We therefore explored text message construction from a semiotic perspective (ie, an analysis of how meanings are created) in order to improve our understanding of the meaning that participants would actively construct from the evidence-based hypertension management messages transmitted to them. During focus groups, we invited participants to discuss their interpretation of content of the message. This included their perception of potential positive and negative characteristics of each message based on their knowledge of the sociocultural realities and perspectives within their community.

In addition to exploring the meaning that participants created from the messages, we were also interested in how the communication of advice in each message would influence the message receiver’s behavior. To conceptualize the basic conditions that influence behavioral change, we applied the behavioral change wheel (BCW) as our theoretical model to explain the relationship between the emerging themes in our qualitative research and health behaviors required for hypertension management [21]. Briefly, the BCW is a theory- and evidence-based tool designed to analyze the nature of health behaviors as well as the mechanisms, supports and policies required to bring about behavioral change. We focused on the 3 essential internal and external conditions of the behavior system necessary to bring about behavioral change in the BCW. These conditions are capability, opportunity, and motivation [21]. The qualitative researchers on this project integrated conversational prompts to inquire about the capability, opportunity, and motivation of community members to follow the advice provided by each of the messages. In focus group discussions, the terms capability, opportunity, and motivation were used consistently with descriptions used in the theoretical model of the BCW [21]:

Capability is defined as the individual's psychological and physical capacity to engage in the activity concerned. It includes having the necessary knowledge and skills.

Opportunity is defined as all the factors that lie outside the individual that make the behavior possible or prompt it.

Motivation is defined as all those brain processes that energize and direct behavior, not just goals and conscious decision-making. It includes habitual processes, emotional responding, as well as analytical decision-making.

To explore these essential conditions from the participants’ perspective, focus group facilitators invited a dialogue about (1) the participants’ beliefs about the meaning of the health behavior messages (and if it matched or diverged from the scientific evidence), (2) how well participants perceived the behaviors suggested in the messages to be accepted within their community, and (3) if potential participants would have sufficient control over their circumstances and motivation to enact the suggested behavior.

In our discussions, we also explored the concept of cultural safety. Cultural safety encompasses reflection on social, political, and historical contexts to health care and an awareness that power relations need to be addressed to improve health and that science and medicine are laden with culture [22-23]. We invited focus group participants to explore these concepts in relationship to the suggested behaviors in the messages during the focus groups. This concept helped to expand our engagement with the concept of culture in DREAM-GLOBAL from a simplistic listing of cultural differences to an examination of social processes and actions that influence inequities in health.

### Ethics

The study is based on community-based participatory research and had to be formally presented to, and approved by decision-making bodies in all participating communities through Village Council approvals in Tanzania and Band Council Resolutions in First Nations in Canada. The study protocol was reviewed by the following university and community-based research ethics review boards: The National Institute for Medical Research in Dar el Salaam, Tanzania (approved March 19, 2014); Queen’s University Health Sciences and Affiliated Teaching Hospitals Research Ethics Board, Kingston, Ontario (DMED-1603-13 approved June 21, 2013); Sunnybrook Health Sciences Centre Research Ethics Board, Toronto, Ontario (#182-2013, approved May 31, 2013); the Cree Board of Health and Social Services of James Bay, Ontario (approved September 11, 2013); Manitoulin Anishinaabek Research Review Committee (MARRC), Ontario (approved October 7, 2013).

## Results

Four focus group sessions were conducted, with a total of 45 participants (see Table 1).

**Table 1:** Focus group sessions.

Focus group	Number of participants	Gender of participants	
		Female	Male
First Nation A	12	9	3
First Nation B	10	8	2
First Nation C	8	4	4
Tanzanian Village A	15	10	5
Total	45	31	14

Our qualitative analysis focused on the identification of major factors that may mediate the effectiveness of the transmission of the content of evidence-based health knowledge and therefore result in divergent or shared meaning between the sender (the researchers) and the recipient (the research participants) of messages. The focus group transcripts provided sufficient data to reach saturation of categories. The analysis revealed that the main categories of these mediators were discussed in the First Nations focus groups in Canada as well as in Tanzania, although some distinctions were noted within subthemes. The major thematic categories of the impact experienced by participants in response to messages are discussed below.

### Feeling Persuasiveness in Positively Framed Advocacies

Participants in all focus groups repeatedly stressed that a positively framed message was more persuasive than negatively framed messages. Discussing a message that instructed patients to “keep taking their medication as instructed”, one participant noted:

There’s no positive (in this message). You need a positive first. Even though you are having a bad day (in terms of hypertension management), it should still be something in regards to saying well maybe you can do better. Say: hope you’re having a good day. Have a good day and don’t forget to take your pills. (The message needs to be) encouraging and positive.

First Nation Community A

For those messages where positive and negative aspects are discussed, such as the benefits of pharmacotherapy and the drawback of potential side effects, participants felt the order of the information is important:

Maybe reverse the message—talk about the benefits before you talk about the symptoms. Because you are putting the bad before the good, maybe you should put the good before the bad—people would respond a bit better.

First Nation Community A

Messages sent to participants in response to healthy blood pressure readings within the desired range were thought to best provide positive personal feedback such as “You are doing a good job of managing your blood pressure!” Participants perceived this kind of positive and affirming feedback as a key motivating factor:

This would encourage me. It would say “Something is working for me!”

First Nation Community B

Participants in Tanzania also stressed the importance of understanding the linguistic variations of the Swahili dialect in the region to avoid words with local negative connotations when crafting messages. This is particularly important when explaining side effects of medication.

Certain words might have a very negative connotation which was not intended, for example the word “dalili” means: “a negative effect… it will harm you”. It also means “symptoms”, but it has a very negative meaning. It is better to exchange it with “madhara” which means “mild effect”.

Tanzanian Village A

### Experiencing Incapacitation in Response to Fear- or Stress-Inducing Messages

Participants in all focus groups expressed the belief that life in their communities subjected them to a lot of stress because many day-to-day necessities were beyond their direct control. One message specifically designed to inform patients of the heart relaxing properties of medication was regarded with skepticism. A participant questioned how this medication could possibly work if patients still feel stress after taking their medication. This may be pointing to a more holistic view of health compared with western medicine, a view which closely links emotional discomfort such as stress with physical disease:

How can (the medication) make you more relaxed when you are always worried about something?

First Nation Community C

Participants also asked that care should be taken not to unduly alarm people with fear-based messages which would cause even more stress. For example, the phrasing of messages to act urgently on a high blood pressure reading prompted participants to caution that community members may not be able to act quickly due to a lack of access to services. Instructions to hurry may therefore be counterproductive:

It would scare me—“you have to hurry” (to have your blood pressure checked and managed)”. I know it’s a fact, they need to scare you. But if somebody’s scared it will just freeze them up, (make them) panic.

First Nation Community B

Similarly, sternly worded warning messages about skipping medications were also perceived as stress-inducing. Instead, reminders were preferred:

If you get busy and you forget then this message gets you worried. It would flip me out. Better to say: “did you take your meds today?”

First Nation Community C

Furthermore, fear-inducing words like “bad outcomes” in English or “kapooza” in Swahili (a graphic description of stroke in the Swahili language) were also discouraged as participants believed it would simply scare people instead of encouraging positive health behaviors:

What about removing the word “bad” and just say “outcomes such as stroke”. Everybody knows stroke is bad. It’s too scary (otherwise).

First Nation Community B

Take the word” kapooza” out and put just “stroke”—everyone understands that (English) word because in the past there was no Swahili word for stroke.

Tanzanian Village A

### Growing Resistance in Response to Oppressive or Authoritarian Messages

Historically, western medicine is a foreign medical system in the DREAM-GLOBAL target communities, who practiced their own forms of traditional medicine. In Canada in particular, where there is a history of colonialism and oppression of Aboriginal people, their traditions, and medical practices by the western government, we heard in discussion that pharmacotherapies still symbolize this hegemony for some people.

It is really hard for the elders to understand why they have to take pills. They have a lot of qualms about pill taking. But some just take whatever they are told.

First Nation Community C

Others are not just apprehensive, but suspicious that the western medications are intended to be harmful. This fear was articulated to be related to the experience people or their family members had with the Indian Residential School (IRS) system throughout the Canadian colonial history. The IRS system was operating in Canada from 1831 to 1996 and has touched most Aboriginal families [24]. To attend IRS, Aboriginal children were often forcibly removed from their parents and subjected to harsh and often abusive treatment during their stays at the IRS [25]. This history is embodied in the explanation offered by one of the First Nations participants:

It is really hard for older people to accept medication; they are so set in their ways. Some never do adapt. They develop ideas, so and so is giving me those pills because they (non-Aboriginal people) want to kill me. They have things on their minds, their own views, (like) “If you try to scare me I will stop taking my pills.”

First Nation Community C

The oppressive relationship between the Canadian government and Aboriginal people has infiltrated many aspects of their daily life and is historically deeply intertwined with the medical system [25-26]. As a consequence, active resistance may become the response to instructions that are perceived as authoritarian:

Ask: “Did you take your medication?” instead of saying “Take your medication!”… Or send a simple reminder…It is less intrusive. Otherwise you are telling me what to do! But you can’t tell me what to do. I am taking it when I am ready to do it…you get tired of listening to this [authoritarian approach from outsiders].

First Nation Community C

Although oppression was less of a discussion topic in Tanzania, this concept nevertheless emerged also in conversations with Tanzanian participants. For example, when participants reviewed the message ‘keep taking your medication as you have been instructed!’ the authoritarian tone was also challenged:

When you translate the word “instructed” (in this message), use the Swahili word “endelea”. It means “keep on going (with this medication)”. It feels more like a reminder. It is the most polite way of saying this. The direct translation of the word “instructed” is a word that is more of a command, which we are not using (in friendly conversation)…

Tanzanian Village A

### Feeling Empowered by Messages That Build on Healthy Cultural and Traditional Practices

Just as important as staying away from authoritarian or oppressive messaging, participants suggested building on strengths within their culture whenever possible by reinforcing healthy cultural and traditional practices.

For example considering alternative flavorings for sodium, Tanzanian participants suggested that herbs were not used in their cooking but there were alternatives that could be considered:

We don’t have other spices we can add. The alternatives to salt include: a sauce of pepper or tomato.

Tanzanian Village A

Aboriginal participants cautioned against blanket statements about the reduction of meat in their diet, as many of their diets were based on wild meats which are much lower in fat than meat from domesticated animals. Therefore suggesting increasing traditional meats compared with domesticated meat consumption was perceived as the better message.

Lean meat - what about moose meat? Moose meat is excellent. There’s two Canada Food Guides—there’s the Native Canada Food Guide. Part of that is moose meat—it has less fat. That is a traditional aspect.

First Nation Community A

Participants also stressed that some foods were not only part of traditional life but also an important food staple acquired through hunting; therefore a change in some food items may simply not be possible for people at this time due to food preferences and the prohibitively high cost of alternatives.

Don't change traditional meat eating patterns.

First Nation Community B

Don’t say to avoid smoked foods. People eat that…We use smoked, we like smoked fish—that’s our traditional food.

First Nation Community B

Physical activity was also discussed in its traditional form, such as “walking in the bush” to pick plants or hunt and participants explained that these traditional land-based activities could also be excellent for stress reduction. Others stressed the importance of incorporating messages encouraging traditional activities over modern Western concepts of fitness such as fitness center based workouts or fitness classes.

(Add) snowshoeing, canoeing, cross country skiing (to your physical activity messages).

First Nation Community B

### Ability to Adopt Specific Health Behaviors Is Shaped by Local Social Determinants of Health

Participants in both countries stressed that the messages should be centered around local realities such as income, education and cultural norms: essentially those that make up the social determinants of health (SDOH). The need to consider the generally low income, barriers to access services and foods, and cultural preferences for food and exercise were particularly emphasized.

In many Aboriginal communities in Canada, fresh fruit and vegetables were described as simply not affordable and many varieties are therefore not realistic as food choices.

Not everybody eats fruit every day. They are too expensive for one thing.

First Nation Community B

I know a lot of poor people that use (Food Banks) and can’t afford to buy bananas, apples and oranges. They eat meat and potatoes and peas and that’s it.

First Nation Community A

The experience in Tanzania was quite similar to the Aboriginal experience:

We eat fruits—in season—but when they are not in season it is difficult.

Tanzanian Village A

Further, in Tanzania, participants advised not to send messages to reduce meat consumption because meat was very expensive and meat consumption was therefore already very low:

Don’t say: “Reduce the amount of meat you eat per sitting.” We don’t normally eat a lot meat because we can’t afford it. (1/2 kilo for the whole family).

Tanzanian Village A

In other cases, participants stressed to only use geographically relevant messages. For example for people living inland in rural Tanzanian villages, fresh fish is not affordable. Only salt cured fish can be purchased:

Change (the messages about fish) to avoid salty fish. None of the other (messages about the health benefits of fish) apply.

Tanzanian Village A

In Tanzania, exercise advice was also adapted to include only those that are culturally relevant.

Eliminate hiking, bicycling, swimming and fitness classes. Instead say: walking quickly, digging, chopping firewood when done regularly will help to reduce your blood pressure.

Tanzanian Village A

In Canada, the diversity of Aboriginal communities also required further tailoring of physical activities to ensure relevance to climate, access and affordability.

### Health Literacy Is Supported by Pragmatic Messages That Fit the Local Context

In terms of phrasing, participants discussed that health messages would need to be clear and practical and describe easily actionable advice like “rinse your canned beans to remove extra sodium” instead of higher level information such as “for better heart health reduce sodium in your diet.” The second message assumes that the patient has the correct health knowledge about hidden sodium in their diet, which may not be accurate.

Similar discussion ensued around practical tips for relaxation and stress reduction. In Canada participants requested clear instructions on techniques that stimulate relaxation and in Tanzania participants suggested an emic term to describe relaxation, which in that cultural context requires first a calm mind before the body can be relaxed:

Give examples, like: “try deep breathing” or “have you gone for a walk today?”

First Nation Community A

Change message to “try to have peace of mind to help control your high blood pressure”.

Tanzanian Village A

Clarity was also requested for accessing health care providers for high blood pressure and what to do in the meantime as one is waiting to access services:

So where do you go when (your blood pressure) is high? ...what can you do for yourself so you don’t have to go to the emergency room?

First Nation Community C

In some cases suggestions for particular designations to describe the health care providers were made to ensure community members would be clear on who the patient is to contact. In Tanzania, participants decided that a title was best:

No problem with the word “daktari”. People take this to mean anyone at the clinic.

Tanzanian Village A

Clear and practical instructions were also sought for physical activity.

Add the kinds of exercise (recommended to improve blood pressure)—be specific.

First Nation Community A

Finally there was consistent emphasis that when the Indigenous languages are used, testing with the local population is required to ensure the appropriate dialect is employed as considerable variation exists in the meaning of some words or phrasing in different geographic areas. The discussion points demonstrate the importance of pragmatic content to enable participants to follow instructions and improve health behaviors.

### Summary of Themes and Subthemes

Our research uncovered six main themes or factors that influence the level of congruence between the message content that researchers perceive to pass on to message recipients versus the message content that is actually perceived by message recipients. Based on these substantive theories, Table 2 provides operationalizing strategies for the development of text messages in order to optimize the congruence of the perceived message content between sender and recipient. These strategies are based on our analysis of themes and subthemes and their relevance to message development.

We then checked for alignment of the main themes and operationalizing strategies with the BCW essential conditions of behavioral change (ie, *capability, opportunity*, and *motivation)* in order to determine the fit of our findings with this theoretical model of behavioral change [21].

We found that each of the 6 main themes and their related operationalizing strategies are a close fit with one or more of the BCW`s three conditions of the behavior system. For example, positively framed messages appear to be acting on message recipients’ motivation for behavioral change, whereas recognizing SDOH would impact on recipients’ capability and opportunity for behavioral change. The relationship of the behavioral change conditions to each of the main themes is provided in Table 2.

**Table 2:** Main themes and operationalizing strategies for text message development.

Main strategies based on themes	Operationalizing strategies for message development based on subthemes	Behavioral change wheel
Use positively framed advocacies, they are more persuasive; avoid negative or non-affirming framing of advocacies	Empower and ease stress by pointing to successes	Motivation
	Inspire	Motivation
	Show respect for receivers	Motivation
	Show compatibility with positive indigenous views of health as “living a good life”	Motivation
Avoid fear- or stress-inducing messages	Do not exacerbate people’s stressful lives (eg, experience of low income or racism)	Motivation
Avoid oppressive or authoritarian messages	Show respect for autonomy: Authoritarian messages are perceived as lacking in respect; invoke historic distrust issues with colonial/medical system; and may cause defiant response	Motivation
	Provide healthy life style education message along with pharmacotherapy	Motivation
Build on healthy cultural and traditional practices whenever possible; avoid incongruity with cultural and traditional practices	Empower with a strengths-based approach to local culture	Capability, opportunity, motivation
	Show respect for culture	Capability, opportunity, motivation
Recognize social determinants of health as drivers of ability to adopt behaviors; avoid disconnect with the reality of social determinants of health and the diversity of cultures within a population	Consider cultural settings and cultural norms related to lifestyle	Capability, opportunity
	Understand affordability and accessibility of foods and medications	Capability, opportunity
	Consider access to providers and/or medications in the health care system	Capability, opportunity
Ensure pragmatic content within the local setting ; avoid lack of clarity and lack practicality of content	Preference for practical tips over higher level advice	Capability
	Avoid ambiguity in wording and assumptions	Capability
	Consider and check the local dialect in translation	Capability

## Discussion

### Framework for the Development of Culturally Safe Messages

Inherent in the development of SMS text messages for mobile health interventions is a collection of (often tacit) assumptions about the function of the text messages and in particular the way in which they may impact on health behaviors. Our research provides a detailed examination of the underlying behavioral theories and their mechanism of interaction with our constructed grounded theory to explain how the content and meaning of text messages is abstracted by the receivers. We found that the meaning received can differ significantly from the meaning perceived by the sender and as a result mediate the receiver’s health behaviors in previously unanticipated ways.

Semiotic approaches to meaning help us understand how meaning is derived in communication beyond the meaning of simple text, through more abstract, tacitly embodied signs and symbols. These symbols may not be shared between the sender and the recipient. Lotman explores text as a meaning-generating mechanism from a semiotic perspective as follows:

...the everyday receiver of information is concerned with the content of the message...the text is treated as something not valuable, not in itself, but merely as a kind of packaging from which the topic of interest is extracted [27]

The function of this process of information exchange in health communication then is to transfer the message in such a way that the thought and meaning envisioned by the sender (or researcher) matches the thought of the receiver (or participants) as they decode the message as much as possible. Lotman explains that:

the system works well if the message received by the addressee is wholly identical to the one dispatched by the addresser and it works badly if there are differences between texts. The differences are classed as errors [27]

**Figure 1:**
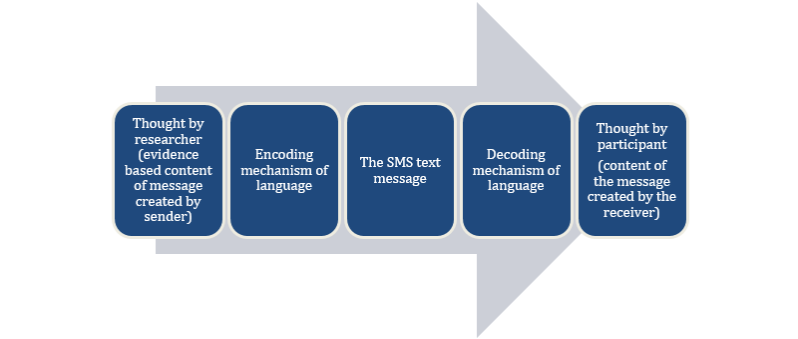
Generation of meaning from SMS messages in mobile health interventions.

We applied Lotman’s concepts to SMS message development in order to deconstruct how meaning is generated in text messages for health interventions such as DREAM-GLOBAL and to identify the point where errors are most likely to occur (see Figure 1). We postulated that the decoding stage can result in differences (or errors) if the factors that mediate the perspectives of the receivers during the decoding stage are poorly understood by the message creators.

**Figure 2:**
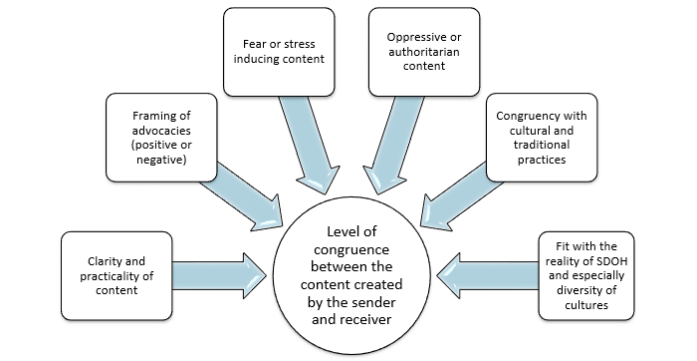
Grounded theory of mediators of meaning in text messages in mobile health interventions in Aboriginal communities in Canada and rural villages in Tanzania.

Conversely then, these errors can be significantly reduced through rigorous research of the factors that mediate decoding and applying this knowledge in the encoding process. Major factors that mediated decoding and the creation of meaning among Aboriginal populations in Canada and rural populations in Tanzania based on our constructed grounded theory are illustrated in Figure 2. We have named these factors the Mediators of Meaning. Operationalizing strategies to apply the Mediators of Meaning in the encoding process (ie, the crafting of SMS messages) are provided in Table 2.

**Figure 3:**
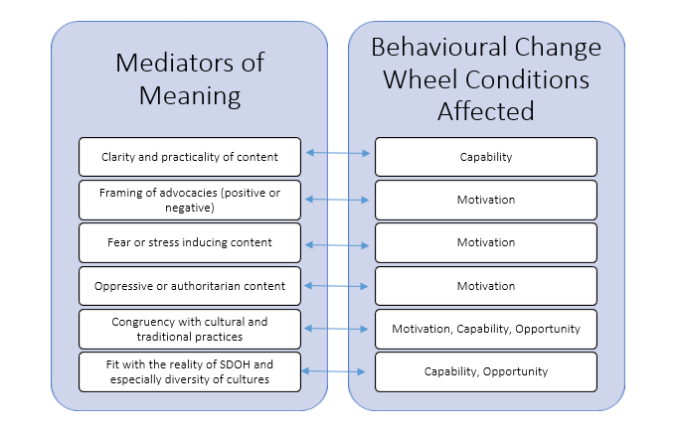
BCW conditions impacted by the mediators of meaning in SMS messages.

The significance of our grounded theory of mediators of meaning is further underscored by their fit and influence on the conditions of capability, opportunity and motivation which are essential to the creation of health behavior change according to the BCW. This finding is particularly significant as all mobile health interventions are arguably always designed to change one or more components in this behavior system. Figure 3 illustrates the behavioral conditions that are most impacted by each of the mediators of meaning identified in our research.

### Implication of Our Research for the Development of Text Messages for Health Interventions

We created a formative research process to develop culturally-based and theory-driven text messages from a set of guidelines based SMS messages to support lifestyle and medication management for hypertension prevention and management. We employed a process that involved close collaboration and dialogue between researchers and representatives of the intended group of recipients of messages. This collaboration focused on developing congruency between the message content sent by the researchers and the message content received by the target population.

Our approach has gone beyond simplistic tailoring of messages to different cultural settings. It allowed us to unpack the hidden assumptions related to culture, health and the health system, and tacit explanatory models of hypertension with which health communication text might be laden. Instead, our methodology provided space for a dialogue between external experts with technical health knowledge and community experts with the lived experience understanding. This dialogue allowed us to draw conclusions about why and how culture and context mediate meaning and to explore the phenomenon of errors of meaning in the transmission of health messages. Our research approach was highly effective in the development of appropriate content and acceptable messages for hypertension management and prevention in diverse cultural settings. Based on our research, we are confident that our approach to the development of theory-driven, evidence-based, and culturally safe text messages in mobile health interventions could be adapted to other cultural and geographic environments and various health issues. Our approach can be used as a model to test and adapt text messages in diverse mobile health intervention projects within various cultural contexts. It can also be applied to health communication message development more generally.

Our constructed grounded theory of the six major mediators of meaning that impact directly on the conditions necessary for behavioral change have served as an effective framework for message development in four different settings where target populations face diverse economic, geographic and structural disparities. The congruence between these mediators and behavioral change theories (see Figure 3) is another indication of the potential applicability of these mediators as a guiding framework to the development of text messages in various mobile health interventions, particularly in low resource environments. The strategies outlined in Table 2 should therefore be considered and tested in the development of text messages in other mobile health interventions. To further substantiate our model, future research should also directly focus on how participants respond to text messages developed with this approach and consider further exploring gender as a mediator as part of the SDOH category.

### Limitations

The SMS messages have been successfully implemented in six First Nations communities in Canada with positive informal feedback on the acceptability of the content from Aboriginal stakeholders during the currently ongoing process evaluation of the DREAM-GLOBAL pragmatic randomized controlled trial (RCT). However, the trial is still in its early implementation phase in Tanzania and conclusive data on retention or outcomes is not yet available. Additional formal evaluation research on the effectiveness of our approach to optimize mediators of meaning in our SMS messages is required to further substantiate this model of formative research for mobile health interventions.

### Conclusion

Our research shows how discrepancies develop between the message created by researchers and the message received by the recipient in mobile health interventions. In our grounded theory research involving Aboriginal people from Canada and rural villagers in Tanzania, the discrepancies between researchers and recipients were primarily generated by six mediators of meaning in SMS messages, including (1) negative or non-affirming framing of advocacies, (2) fear- or stress-inducing content, (3) oppressive or authoritarian content, (4) incongruity with cultural and traditional practices, (5) disconnect with the reality of the SDOH and especially the diversity of cultures within populations, and (6) lack clarity and/or practicality of content.

The six mediators of meaning provide the basis for sound strategies for message development because they impact directly on the necessary conditions of behavioral change of the BCW. These differences between the sent and received messages impact on behavioral changes by acting directly on capability, opportunity and motivation for behavioral change. This kind of critically-oriented formative research is necessary to develop messages that are not only evidence-based but also congruent with the target populations’ capabilities, opportunities, and motivations, in order to optimize the necessary conditions for improvements in health behaviors. Outcome research after implementation of the intervention regarding the acceptability of the messages as perceived by users is needed to further substantiate our model.

Our research underscores the urgent need for mobile health interventions to incorporate and evaluate the quality of SMS messages and to examine the mediators of meaning within each cultural and demographic target group, because the quality of text messages impact significantly on the effectiveness of a text message-based health intervention. Reporting on formative research will improve the transparency in mobile health intervention research and allow researchers in the field to move away from the current black box of SMS text message development.

## Abbreviations

BCW: behavioral change wheel
CBPR: community-based participatory research
CHEP: Canadian Hypertension Education Program
DASH: dietary approaches to stop hypertension
DREAM-GLOBAL: Diagnosing hypeRtension - Engaging Action and Management in Getting LOwer Bp in Aboriginal and LMIC
IRS: Indian residential school
LMIC: low- and middle-income countries
RCT: randomized controlled trial
SDOH: social determinants of health
SMS: short message service
